# Irrational Beliefs and Their Role in Specific and Non-Specific Eating Disorder Symptomatology and Cognitive Reappraisal in Eating Disorders

**DOI:** 10.3390/jcm10163525

**Published:** 2021-08-11

**Authors:** Lucia Tecuta, Valentina Gardini, Romana Schumann, Donatella Ballardini, Elena Tomba

**Affiliations:** 1 Department of Psychology, University of Bologna, 40127 Bologna, Italy; lucia.tecuta2@unibo.it (L.T.); valentina.gardini8@unibo.it (V.G.); 2 Centro Gruber, Eating Disorders Outpatient Clinic, 40125 Bologna, Italy; r.schumann.gruber@gmail.com (R.S.); donatella.ballardini@yahoo.com (D.B.)

**Keywords:** cognitive–behavioral therapy, eating disorders, maladaptive cognitions, irrational beliefs, cognitive reappraisal

## Abstract

Background: Research on which specific maladaptive cognitions characterize eating disorders (ED) is lacking. This study explores irrational beliefs (IBs) in ED patients and controls and the association between IBs and ED-specific and non-specific ED symptomatology and cognitive reappraisal. Methods: 79 ED outpatients with anorexia nervosa, bulimia nervosa, or other specified feeding or eating disorders and 95 controls completed the Attitudes and Beliefs Scale-2 (ABS-2) for IBs. ED outpatients also completed the Eating Disorder Inventory-3 (EDI-3) for ED-specific (EDI-3-ED Risk) and non-specific (EDI-3-General Psychological Maladjustment) symptomatology; General Health Questionnaire (GHQ) for general psychopathology; Emotion Regulation Questionnaire (ERQ) for cognitive reappraisal. Results: Multivariate analysis of variance with post hoc comparisons showed that ED outpatients exhibit greater ABS-2-Awfulizing, ABS-2-Negative Global Evaluations, and ABS-2-Low Frustration Tolerance than controls. No differences emerged between ED diagnoses. According to stepwise linear regression analyses, body mass index (BMI) and ABS-2-Awfulizing predicted greater EDI-3-ED Risk, while ABS-2-Negative Global Evaluations and GHQ predicted greater EDI-3-General Psychological Maladjustment and lower ERQ-Cognitive Reappraisal. Conclusion: Awfulizing and negative global evaluation contribute to better explaining ED-specific and non-specific ED symptoms and cognitive reappraisal. Therefore, including them, together with BMI and general psychopathology, when assessing ED patients and planning cognitive–behavioral treatment is warranted.

## 1. Introduction

The literature supports the clinical utility of cognitive–behavioral therapy (CBT) models and therapies in many psychopathologies [1], and they are often considered first-line treatment in mental health care [2,3]. However, empirical studies on maladaptive cognitions, which are a key target in this approach, and their role in psychopathology are scarce.

Within the expansion of CBT variants, a type of maladaptive cognitions, irrational beliefs (IBs), have long been hypothesized to play a role in all psychopathologies and are a central target of Ellis’ Rational Emotive CBT model [4]. In this model, four types of IBs have been clinically and theoretically identified: negative global evaluations of the self and others, awfulizing thoughts (excessive negative evaluations and expectations of events), low frustration tolerance beliefs (the impossibility to tolerate an event or set of circumstances), and demandingness (rigid expectations of the self and others). So far, only one meta-analysis [5] has empirically supported the association between IBs and various types of psychological distress (including anxiety, depression, anger, and guilt), while their role in specific psychopathologies needs further experimental investigation.

Research on IBs might be particularly relevant in eating disorders (EDs), as there is a need to improve CBT treatment retention and outcome rates in this population [1]. While cognitive features such as preoccupations regarding food, weight, and shape have been widely investigated in EDs, only a few studies examine other cognitively oriented conceptualization of maladaptive cognitions beyond strictly ED-related themes in this population [6,7,8,9]. Broadening the cognitive targets in EDs may help to better represent different aspects of the cognitive processes associated with the ED psychopathology, which include related general psychopathology, in addition to ED-specific symptomatology [10]. Non-specific ED symptoms such as low self-esteem, reduced interoceptive awareness, and affective problems have been recently discovered to have a central role in EDs in studies using network analysis, a promising method to reconceptualize EDs [10].

To the best of our knowledge, one study [11] has investigated IBs in an ED mixed sample, albeit using a global score of IBs in relation to body dissatisfaction. In another, the relationship between a global level of IBs and psychopathological features such as general psychological maladjustment (e.g., perfectionism, low self-esteem, and interpersonal difficulties) that characterize EDs has been supported as well [12]. The global level of IBs also resulted in being associated with other clinical dysfunctional features known to have a role in the maintenance of EDs [13] such as impaired emotion regulation strategies in particular cognitive reappraisal, the capacity to alter one’s emotional state by cognitively reassessing the situation [14]. Considering that CBT therapies—first line treatment for EDs—promote cognitive reappraisal through cognitive restructuring and behavioral exposure techniques as a primary mechanism of cognitive change [4,15,16], recognizing empirically such an association has important clinical implications.

Less is known about which specific types of IBs may characterize specific ED diagnoses and which specific IBs may have a role in ED core symptomatology, non-ED-specific psychopathology, and cognitive reappraisal. Such an identification is warranted, since several authors have indeed called for further research aimed at identifying which particular cognitive targets might contribute to better ED outcomes [17,18,19] and at offering additional empirical support for the mechanisms underlying widely used ED cognitive models [20,21].

The present study aims at examining differences in specific IBs between ED patient diagnostic subgroups and general population controls as well as exploring the contribution of specific IBs on in ED-specific symptomatology, non-ED-specific psychopathology, and cognitive reappraisal. It is expected that IBs will be more pronounced in ED patients compared to controls and that IBs will predict greater ED symptomatology, ED-related psychopathology, and greater cognitive reappraisal difficulties.

## 2. Materials and Methods

### 2.1. Participants

The ED group included consecutively screened outpatients (*n* = 82) who met Diagnostic and Statistical Manual of Mental Disorders 5 criteria for EDs [22] (DSM 5) anorexia nervosa (AN), bulimia nervosa (BN), and other specified feeding or eating disorder (OSFED). Patients were contacted from a specialized ED treatment center before commencing CBT-based treatment with integrated nutritional rehabilitation and invited to take part in the study. ED diagnoses were established at intake by the consensus of a psychiatrist and a clinical psychologist independently using the Structured Clinical Interview for DSM 5 (SCID-5) [23]. With the exception of three patients who refused to participate, all invited patients took part in the study (*n* = 79). The inclusion criteria were: (a) 18 to 65 years of age, (b) a diagnosis of AN, BN, or OSFED, (c) within one month of beginning treatment. The exclusion criteria were: (a) lack of capacity to consent for research, (b) ED diagnosis secondary to a physical health or metabolic condition, (c) comorbid drug/alcohol abuse, psychotic or neurocognitive disorders, acute suicidality, and pregnancy.

Control participants matched for gender and age were recruited from the adult general population online and on university campuses in Northern Italy with the following inclusion criteria: (a) 18 to 65 years of age and (b) no prior diagnosis of any ED according to DSM 5 diagnostic criteria. Exclusion criteria were: (a) lack of capacity to consent for research and (b) lifetime history of EDs according to DSM 5 diagnostic criteria, either as primary diagnosis or in comorbidity to other mental health and physical conditions. The project was approved by the University of Bologna Bioethics Committee and Department of Psychology Ethics Committee. Informed consent was obtained from all participants included in the study.

### 2.2. Measures and Clinical Variables

Outpatients before commencing treatment and controls were assessed through the following self-rating questionnaire:

(1) The Attitudes and Beliefs Scale 2 [24] (ABS-2) is composed of 72 Likert scale items, which can be classified according to four IBs, which are demandingness, awfulizing, low frustration tolerance, and negative global evaluations. Items of the ABS-2 can also be classified across three types of contents including approval (“If loved ones or friends reject me, it is not only bad, but also the worst thing that could happen to me”), achievement (“I must do well at important things, and I will not accept not doing well”), and comfort (“It is unbearable to feel uncomfortable, tense, or nervous and I cannot stand it when I do”). Negative global self-evaluations refer to generalized negative labeling and self-statements. Demands represent rigid, inflexible, and nonpragmatic beliefs and reflect absolutistic “must statements”. Awfulizing statements are instead excessive negative evaluations of events, while low frustration tolerance beliefs refer to thinking that one cannot tolerate an event or set of circumstances. The Italian translation was used with excellent internal consistency of the measure (α = 0.926), as well as for total and subscale scores. Cronbach α coefficients have been reported with 0.738 for demandingness, 0.759 for awfulizing, 0.832 for low frustration tolerance, and 0.810 for negative global evaluation/self-downing [19].

The clinical sample of outpatients was also assessed using the following interviews and self-rating questionnaires:

(2) The Emotion Regulation Questionnaire [25] (ERQ) is a 10-item questionnaire that assesses emotion regulation strategies of expressive suppression and cognitive reappraisal. The 10 items composing the ERQ are scored on a 7-point Likert scale according to the level of agreement with the reported sentence (1 being complete disagreement and 7 being complete agreement). The ERQ is composed of two subscales: Cognitive Reappraisal and Expressive Suppression, comprising six items and four items, respectively. Validation studies showed that both subscales have an adequate internal consistency, as well as in the Italian version [26] where Cronbach’s alpha scores were 0.84 for the reappraisal scale and 0.72 for the suppression scale. Only the cognitive reappraisal scale was used in the current study.

(3) The Eating Disorder Inventory-3 [27,28] (EDI-3) is a self-rating 91-item questionnaire assessing clinically relevant psychological traits and constructs in EDs. It yields six composite scales two of which were used in the study: eating disorder risk/concerns and general psychological maladjustment. The ED risk/concerns scale refers to ED-specific symptomatology and includes scales of bulimia, drive for thinness, and body dissatisfaction. The general psychological maladjustment scale represents a total global psychological functioning index and indicates levels of ED-related psychopathology. It is a composite score of the nine psychological trait scales of the EDI-3 including low self-esteem, personal alienation, interpersonal alienation, interpersonal insecurity, interoceptive deficits, emotion dysregulation, perfectionism, ascetism, and maturity fears.

(4) The General Health Questionnaire—30 item version [29] (GHQ-30) is an instrument aimed at evaluating depressive and anxiety symptoms, sleeping problems, social functioning, well-being, and coping abilities. A composite global score is used. Higher scores reflect greater impairment in mental health. Cronbach’s alpha coefficients for the GHQ-30 tested in various empirical studies in community samples ranged from approximately 0.82 to 0.93. Test–retest reliability coefficients varied from 0.50 to 0.90. In this study, the Italian version was used [30].

(5) Baseline body mass index (BMI) and illness duration in months were collected at intake from a medical nutritionist specialized in EDs (D.B.) and recorded in the patient’s medical record before commencing treatment.

### 2.3. Data Analysis

Descriptive statistics were run for socio-demographic and clinical characteristics, and a *t*-test was run to compare EDs and controls in terms of age. A multivariate analysis of variance (MANOVA) adjusted for age and post hoc analyses with Bonferroni corrections was conducted to verify differences between the three diagnostic ED groups (AN, BN, and OSFED) and the control group in the four IBs. Stepwise linear regression analyses adjusted for age, BMI, and baseline GHQ total scores were performed on ED outpatients to determine the contribution of the specific IBs in explaining the variance in EDI-3-ED Risk concerns (ED-specific symptoms) and EDI-3-General Psychological Maladjustment (non-specific ED symptoms), as well as in ERQ-Cognitive Reappraisal. In particular, the specific IBs found to be more significantly different between ED patients and controls were those included in the regression analyses. Prior to conducting a stepwise multiple regression, the relevant assumptions of this statistical analysis were tested.

In all analyses, the level of significance was set at *p* < 0.05 (two-sided). The Statistical Package for Social Sciences (SPSS) was used for all calculations.

## 3. Results

### 3.1. Sample Characteristics

The clinical sample was composed of 79 female ED outpatients with varying diagnoses (37 with AN, 21 with BN, and 21 with OSFED). ED outpatients had a mean age of 26.81 ± 11.78 years. Mean illness duration was 8.58 ± 10.05 years. BMIs for each diagnostic group were as follows: AN (17.27± 2.52 kg/m^2^), BN (21.89 ± 4.68 kg/m^2^), and OSFED (20.58 ± 5.08 kg/m^2^).

Participants of the general population control group were 95 females recruited from the general population and had a mean age of 29.23 ± 10.52 years.

Age did not differ significantly between controls and ED patients (t = −1.431, *p* = 0.154).

### 3.2. Clinical and Control Group Comparisons

There was a statistically significant difference adjusted for age in type of IBs scores among groups as determined by the MANOVA (*F* (12,434.195) = 3.921, *p* < 0.0001; Wilk’s Λ = 0.762, partial η^2^ = 0.087; Table 1). Univariate tests revealed significant differences in awfulizing (*F* (3,167) = 10.307; *p* < 0.0001; partial η^2^ = 0.156), in negative global evaluations (*F* (3,167) = 9.747; *p* < 0.0001; partial η^2^ = 0.149), low frustration tolerance (*F* (3,167) = 8.809; *p* < 0.0001; partial η^2^ = 0.137), and to a lesser extent, in demandingness (*F* (3,167) = 3.163; *p* < 0.026; partial η^2^ = 0.054). More specifically, post hoc tests with Bonferroni adjustments for multiple comparisons revealed that awfulizing scores were significantly higher in AN, BN, and OSFED patients compared to controls. Similarly, negative global evaluation scores were significantly higher in AN, BN, and OSFED patients compared to controls. Low frustration tolerance scores were also found to be significantly higher in AN, BN, and OSFED patients compared to controls. Regarding demandingness scores, no group differences emerged in post hoc tests. Moreover, regarding the ED group, there were no statistically significant differences in all post hoc analyses between ED diagnostic groups in any of the four IBs scales.

### 3.3. Role of Irrational Beliefs in ED-Specific Symptoms, Non-Specific ED Psychopathology, and Cognitive Reappraisal

Multiple linear regression models were developed with ED symptomatology (EDI-3-ED Risk and EDI-3-General Psychological Maladjustment) and ERQ-Cognitive Reappraisal as the dependent variables. Independent variables were the three ABS-2 scales (awfulizing, negative global evaluations, and low frustration tolerance) found to be significantly altered in ED outpatients compared to controls. Analyses were adjusted for age, BMI, and GHQ scores at baseline as an index of severity of general psychopathology.

The results of the stepwise multiple regression analysis indicated that ABS-2-Awfulizing predicted EDI-3-ED Risk scores, with higher awfulizing scores associated with higher ED symptomatology. Moreover, BMI also resulted in being a significant covariate, that is, higher BMI was associated with greater ED psychopathology (beta = 0.326, t = 3.175, *p* = 0.002).

EDI-3-General Psychological Maladjustment was instead predicted by higher scores in ABS-2-Negative Global Evaluations. The covariate of GHQ total scores was also significant, where higher GHQ total scores were associated with greater EDI-3-General Psychological Maladjustment (beta = 0.307, t = 6.429, *p* < 0.0001).

Furthermore, higher scores in ABS-2-Negative Global Evaluations also predicted lower scores in ERQ-Cognitive Reappraisal, adjusting for age, BMI, and GHQ scores at baseline, all of which were excluded from the stepwise procedure. See Table 2 for significant regression models and coefficients.

ABS-2-Low Frustration Tolerance did not appear to be a significant predictor for ED-specific risk/concerns (EDI-3-ED Risk scores), for non-specific ED psychopathology (EDI-3-General Psychological Maladjustment), or for cognitive reappraisal in any of the stepwise regression models.

## 4. Discussion

The current study found that ED outpatients report greater endorsement of all types of IBs when compared to general population controls. This is in line with both cognitive frameworks for psychopathology [15] and cognitive models for EDs [31]. The results also support previous studies suggesting that dysfunctional cognitions, even when stemming from different CBT models, characterize ED psychopathologies [6,9,11]. No significant difference in all types of IBs instead emerged when comparing ED diagnostic subgroups. This lack of significant differences is in accordance with Fairburn’s transdiagnostic model postulating that the main maintaining cognitive processes of EDs are likely to be largely the same across different eating disorder diagnoses [31]. Nonetheless, previous studies found how other conceptualizations of maladaptive cognitions, such as Beck’s core beliefs, did not differ significantly between AN and BN [6]. Similarly, other studies found that Young’s early maladaptive schemas (another way to conceptualize maladaptive cognitions) did not differ between BN and binge-eating disorder (BED) patients [9].

Our study also supports the association between specific types of IBs and different aspects of ED psychopathology, that is, both ED-specific symptomatology and non-specific ED psychopathology and cognitive reappraisal. In particular, the irrational belief of awfulizing (the tendency of negatively evaluating events in absolutistic terms) was found to significantly predict ED-specific risk/concerns, that is, the greater the tendency to awfulize, the greater the ED-specific symptomatology encompassing drive for thinness, bulimic symptoms, and body dissatisfaction. Preoccupations about controlling eating, weight, and shape are widely known to be a main feature of ED psychopathology [30], in which the tendency to catastrophize (a cognitive process similar to awfulizing) is often encountered in CBT clinical practice [15,32]. Along with awfulizing, BMI was found to be the main significant predictor of EDI-3-ED Risk, that is, the greater the BMI the higher the level of ED symptomatology. This is in line with the already well-established association between ED symptomatology and higher body weight, as patients with a higher BMI often also report a greater endorsement of ED symptoms (such as bulimic behaviors and a drive for thinness) in order to lose weight or contrast weight gain [33,34,35]. Not surprisingly, one of the main techniques of cognitive restructuring in all CBT treatment variants for EDs focuses on recognizing and challenging catastrophic thoughts [36], which are mainly related to weight and body shape in ED patients [31]. In one study, AN and BN patients were found to exhibit a greater tendency to catastrophic thinking compared to controls [32]. Additionally, within existing ED cognitive models, significant associations between awfulizing and ED-restrictive and bulimic symptomatology encompassed in the EDI-3-ED Risk scale have also been found. This supports the notion that restrictive and compensatory behaviors are also enacted by ED patients to manage anxious emotional states to regulate mood [2,37,38,39,40]. Moreover, catastrophic thinking and worry are key cognitive factors in anxiety disorders and the hallmark features of generalized anxiety disorder [41], as well as cognitive vulnerability factors in mood disorders [42,43], all of which are psychiatric diagnoses known to often co-occur with EDs [44,45]. Indeed, in some studies, worry emerged as an important feature in dietary control of shape and weight [46,47], and ED patients showed greater endorsement of worry when compared to controls [32].

The irrational belief of negative global evaluations was found instead to be the most significant predictor of non-specific ED general psychological maladjustment, which includes aspects pertaining to perfectionism, self-esteem, inefficacy, and interpersonal difficulties. Feelings of inefficacy in particular have been hypothesized to play a core role in clinical models of Eds, and they have been empirically supported, using network analysis procedures, to reach centrality among nodes of the network of symptoms of ED patients [10,48]. ED patients commonly present low self-esteem, low self-efficacy, and beliefs of incompetence [31,49,50,51,52] linked with ED symptomatology even when considering confounding depressive symptoms [53]. Negative global evaluations or more generally speaking, negative self-beliefs, have also been associated with major depression and depressive symptomatology in the literature [54]. While the presence of depressive disorders was not assessed in the current study, the GHQ evaluation, which is an instrument that also contains items pertaining to depression, was included in the analysis. The GHQ emerged indeed to be a significant covariate contributing to explaining the variance of ED general psychological maladjustment along with negative global evaluations.

The irrational belief of negative global evaluations was found to also have a significant role in explaining the variance of cognitive reappraisal, that is, the greater the endorsement of negative self-beliefs, the lower the ability to reappraise events appropriately. Negative self-beliefs would be associated with compromised cognitive reappraisal, as they represent a form of rigid and biased thinking combined with hypersensitivity to emotionally negative salient information. The presence of negative thinking has been found to be associated with a greater activation of the limbic areas and emotional areas of the brain, which are known to have negative feedback on the pre-frontal cortex [55], which is also in charge of cognitive reappraisal processes. This is supported by studies showing that both ill and weight-restored AN patients engage the medial pre-frontal cortex less than healthy controls for appropriate self-relevant cognitions [56]. This impairment was also found in remitted or recovered ED patients [57], who seem to still exhibit two majorly deficient areas of cognitive functioning: executive functions and emotion regulation processes. Both these cognitive functions encompass cognitive reappraisal skills, which impairment may limit CBT treatment outcomes especially when applying psychotherapeutic ingredients such as cognitive restructuring involving primarily mechanisms of cognitive change [4,15,16]. Moreover, the implementation of cognitive reappraisal, as a type of functional coping strategy to deal with emotions, is particularly important in EDs. In fact, individuals with EDs seem to turn towards pathological ED behaviors as a momentarily beneficial tool to deal with their negative emotions in the absence of healthy and effective strategies, such as the capacity to enact cognitive reappraisal [14].

The discussed findings, which link awfulizing with ED restrictive and bulimic symptomatology and negative global evaluations with non-ED-specific psychopathology and cognitive reappraisal, have important clinical implications. While ED-related maladaptive cognitions [14] concerning food and weight preoccupations are important targets [58], our results support that dysfunctional cognitions regarding the self and unrelated to food and weight [54,59,60] also warrant adequate assessment and careful consideration. These aspects should be included as complementary outcomes while planning CBT treatment.

According to our data, the IBs of awfulizing and negative global evaluations might be considered in CBT-Enhanced (CBT-E) or other cognitive-based treatments of EDs as specifications of the so-called “unhelpful thoughts” [31] that should be targeted particularly in the intermediate phases of treatment [61] following nutritional rehabilitation. Targeting these dysfunctional thoughts could help reducing ED-specific core symptoms and ED-related psychopathology and would be beneficial for cognitive change. In line with the promising method to reconceptualize ED that is network analysis [10], our results support to broaden the core psychopathology to non-ED-specific cognitive symptoms. Including additional psychological cognitive features in the assessment of EDs might yield incremental data to better evaluate and treat the complex interplay characterizing ED psychopathology. This could be done, for example, by measuring levels of specific IBs together with the levels of overvaluation of body shape and weight and of cognitive restraint.

The aforementioned results should, however, be considered in light of several methodological limitations. The relatively small sample size of the included ED diagnostic subgroups lowers statistical power. This issue, together with using a female-only sample, might represent a methodological issue that makes the results not generalizable. Diagnostic group comparisons should, therefore, be repeated in larger ED samples including males and females, and regression analyses should be repeated in non-mixed ED samples. When possible, making comparisons with other clinical populations could further confirm our findings. The use of a cross-sectional research design does not allow us to draw conclusions on the causal relationship between IBs, ED symptomatology and general psychopathology, and cognitive reappraisal. Studies using repeated assessments are necessary to demonstrate temporal precedence between the variables [62]. Future research should investigate longitudinally whether changes in negative global evaluations and cognitions regarding awfulizing may have a role in the mechanisms of change in CBT treatment in EDs. Future research is increasingly needed, as there is a relative lack of understanding regarding which specific mechanisms of change make evidence-based treatments effective [62,63].

## Figures and Tables

**Table 1:** MANOVA and post hoc tests with Bonferroni correction comparing ED patients and controls in types of irrational beliefs (ABS-2).

	Multivariate Tests							
		**Wilk’s Λ**		**F_(df,df error)_**		**Partial η^2^**	***p***	
ABS-2 SCALES		0.762		3.921_(12,434.195)_		0.087	<0.0001	
	**Univariate Tests**					**Post Hoc (with Bonferroni Correction)**		
**ABS-2** **Scales** **(Range)**	**F** **_(df,df error)_**	**Partial η^2^**	***p***	**Diagnostic Groups** **(*n*)**	**M ± SD**	**Pairwise Comparisons**	***p***	**Mean Difference**
ABS-2 Irrational AWF (0–36)	10.307 _(3,167)_	0.156	<0.0001	AN (*n* = 37) BN (*n* = 20) OSFED (*n* = 20) C (*n* = 95)	18.97 ± 8.23 21.40 ± 8.31 19.00 ± 6.80 12.94 ± 7.82	AN vs. BN AN vs. OSFED AN vs. C BN vs. OSFED BN vs. C OSFED vs. C	1.000 1.000 0.002 1.000 <0.001 0.016	−2.900 −0.232 5.682 * 2.670 8.584 * 5.914 *
ABS-2 Irrational DEM (0–36)	9.747 _(3,167)_	0.149	<0.0001	AN (*n* = 37) BN (*n* = 20) OSFED (*n* = 20) C (*n* = 95)	15.62 ± 6.50 17.25 ± 8.14 16.70 ± 5.57 13.35 ± 6.30	AN vs. BN AN vs. OSFED AN vs. C BN vs. OSFED BN vs. C OSFED vs. C	1.000 1.000 0.659 1.000 0.084 0.260	−1.916 −1.202 2.060 0.703 3.976 3.262
ABS-2 Irrational NGE (0–36)	8.809 _(3,167)_	0.137	<0.0001	AN (*n* = 37) BN (*n* = 20) OSFED (*n* = 20) C (*n* = 95)	13.92 ± 10.14 16.25 ± 8.94 14.60 ± 9.42 7.49 ± 7.77	AN vs. BN AN vs. OSFED AN vs. C BN vs. OSFED BN vs. C OSFED vs. C	1.000 1.000 0.003 1.000 <0.001 0.008	−2.895 −9.240 6.004 * 1.970 8.899 * 6.928 *
ABS-2 Irrational LFT (0–36)	3.163 _(3,167)_	0.054	0.026	AN (*n* = 37) BN (*n* = 20) OSFED (*n* = 20) C (*n* = 95)	20.54 ± 6.77 21.30 ± 7.25 21.20 ± 5.06 15.19 ± 7.50	AN vs. BN AN vs. OSFED AN vs. C BN vs. OSFED BN vs. C OSFED vs. C	1.000 1.000 0.003 1.000 0.003 0.006	−1.228 −0.862 5.002 * 0.367 6.230 * 5.863 *

Notes. Analyses adjusted for age. Bonferroni corrections applied. ABS-2, Attitudes and Beliefs Scale 2; AN, anorexia nervosa; AWF, awfulizing; BN, bulimia nervosa; C, controls; DEM, demandingness; ED, eating disorder; LFT, low frustration tolerance; M, mean; MANOVA, multivariate analysis of variance; NGE, negative global evaluations; OSFED, other specified feeding or eating disorder; SD, standard deviation. * Significant at *p* < 0.05.

**Table 2:** Stepwise regression analysis to predict ED-specific symptomatology, ED-related psychopathology, and cognitive reappraisal in ED outpatients.

DV: EDI-3-ED Risk						
Model	IV	B	Std E	Beta	t	*p*
1	BMI	1.767	0.550	0.354	3.212	0.002
2	BMI GHQ	1.713 0.585	0.537 0.272	0.343 0.231	3.187 2.150	0.002 0.035
**3**	**BMI** **ABS-AWF** **GHQ**	**1.625** **0.960** **0.162**	**0.512** **0.328** **0.296**	**0.326** **0.343** **0.064**	**3.175** **2.922** **0.545**	**0.002** **0.005** **0.587**
Model 1: *R*^2^ = 0.125; *Adj* *R*^2^ = 0.113; *F*_(1,72)_ = 10.318; *p* = 0.002 Model 2: *R*^2^ = 0.179; *Adj* *R*^2^ = 0.156; *F*_(1,71)_ = 4.623; *p* = 0.035 **Model 3: *R*^2^ = 0.268; *AdjR*^2^ = 0.237; *F_(_*_1,70)_ = 8.538; *p* = 0.005**						
**DV: EDI-3-General Psychological Maladjustment**						
**Model**	**IV**	**B**	**Std E**	**Beta**	**t**	***p***
1	GHQ	2.478	0.445	0.557	5.572	<0.0001
**2**	**ABS-NGE** **GHQ**	**2.290** **1.364**	**0.356** **0.394**	**0.569** **0.307**	**6.429** **3.466**	**<0.0001** **0.001**
Model 1: *R*^2^ = 0.310; *Adj* *R*^2^ = 0.300; *F*_(1,69)_ = 31.047; *p* < 0.0001 **Model 2: *R*^2^ = 0.571; *AdjR*^2^ = 0.558; *F*_(1,68)_ = 41.327; *p* < 0.0001**						
**DV: ERQ-Cognitive Reappraisal**						
**Model**	**IV**	**B**	**Std E**	**Beta**	**t**	***p***
**1**	**ABS-LFT**	**−0.305**	**0.067**	**−0.484**	**−4.525**	**<0.0001**
**Model 1: *R*^2^ = 0.234; *AdjR*^2^ = 0.223; *F*_(1,67)_ = 20.474; *p* < 0.0001**						

Notes. ABS-2, Attitudes and Beliefs Scale 2; AWF, awfulizing; BMI, body mass index; DV: dependent variable; ED, eating disorder; GHQ, General Health Questionnaire; LFT, low frustration tolerance; IV: independent variables; NGE, negative global evaluations. Final significative stepwise regression models are in bold.

## Data Availability

Due to the nature of this research, participants of this study did not agree for their data to be shared publicly, so supporting data are not available.
